# Lactonase Specificity Is Key to Quorum Quenching in *Pseudomonas aeruginosa*

**DOI:** 10.3389/fmicb.2020.00762

**Published:** 2020-04-24

**Authors:** Benjamin Rémy, Laure Plener, Philippe Decloquement, Nicholas Armstrong, Mikael Elias, David Daudé, Éric Chabrière

**Affiliations:** ^1^Aix Marseille University, Institut de Recherche pour le Développement, Assistance Publique - Hôpitaux de Marseille, Microbes Evolution Phylogeny and Infections, Institut Hospitalo-Universitaire-Méditerranée Infection, Marseille, France; ^2^Gene&GreenTK, Marseille, France; ^3^Department of Biochemistry, Molecular Biology and Biophysics – BioTechnology Institute, University of Minnesota, St. Paul, MN, United States

**Keywords:** quorum sensing, *Pseudomonas aeruginosa*, quorum quenching, acyl-homoserine lactones, lactonases, virulence, biofilm

## Abstract

The human opportunistic pathogen *Pseudomonas aeruginosa* orchestrates the expression of many genes in a cell density-dependent manner by using quorum sensing (QS). Two acyl-homoserine lactones (AHLs) are involved in QS circuits and contribute to the regulation of virulence factors production, biofilm formation, and antimicrobial sensitivity. Disrupting QS, a strategy referred to as quorum quenching (QQ) can be achieved using exogenous AHL-degrading lactonases. However, the importance of enzyme specificity on quenching efficacy has been poorly investigated. Here, we used two lactonases both targeting the signal molecules *N*-(3-oxododecanoyl)-L-homoserine lactone (3-oxo-C_12_ HSL) and butyryl-homoserine lactone (C_4_ HSL) albeit with different efficacies on C_4_ HSL. Interestingly, both lactonases similarly decreased AHL concentrations and comparably impacted the expression of AHL-based QS genes. However, strong variations were observed in *Pseudomonas* Quinolone Signal (PQS) regulation depending on the lactonase used. Both lactonases were also found to decrease virulence factors production and biofilm formation *in vitro*, albeit with different efficiencies. Unexpectedly, only the lactonase with lower efficacy on C_4_ HSL was able to inhibit *P. aeruginosa* pathogenicity *in vivo* in an amoeba infection model. Similarly, proteomic analysis revealed large variations in protein levels involved in antibiotic resistance, biofilm formation, virulence and diverse cellular mechanisms depending on the chosen lactonase. This global analysis provides evidences that QQ enzyme specificity has a significant impact on the modulation of QS-associated behavior in *P. aeruginosa* PA14.

## Introduction

The human pathogen *Pseudomonas aeruginosa* is commonly involved in healthcare associated infections and frequently displays drug or multidrug resistance (Sievert et al., 2013; Weiner et al., 2016). This latter constitutes a serious therapeutic threat. Yet, the quest for new alternative treatments to fight bacterial infections is highly challenging. Disruption of quorum sensing (QS), a cell-density dependent communication system used by this bacterium to regulate the expression of several traits including virulence, has emerged as a non-bactericidal curative approach to address issues of antibiotics resistance (Fuqua and Greenberg, 2002; Bzdrenga et al., 2017; Rémy et al., 2018).

In *Pseudomonas aeruginosa*, three main QS systems have been described namely Las, Rhl, and PQS (i.e., *Pseudomonas* Quinolone Signal) involving the signal synthases LasI, RhlI, PqsABCDEH, and the receptors LasR, RhlR, and PqsR, respectively (Lee and Zhang, 2015; Papenfort and Bassler, 2016). These systems used three signaling molecules referred to as autoinducers. Two acyl-homoserines lactones (AHL) *N*-(3-oxododecanoyl)-L-homoserine lactone (3-oxo-C_12_ HSL) and butyryl-homoserine lactone (C_4_ HSL) are used by Las and Rhl systems while PQS is based on 2-alkyl-4-quinolones (Lee and Zhang, 2015; Papenfort and Bassler, 2016). These systems are interconnected and Las is considered as the global activator of all three systems (Latifi et al., 1996; Déziel et al., 2004).

Interferences with QS are termed *quorum* quenching (QQ) and mainly involve chemical inhibitors (QSIs) or autoinducer degrading enzymes (Grandclément et al., 2016; Rémy et al., 2018). QQ enzymes able to target AHLs belong to three classes: lactonases (EC 3.1.1), acylases (EC 3.5.1) and oxidoreductases (EC 1). Among lactone-degrading enzymes, paraoxonases, metallo-β-lactamase like lactonases (MLL) and phosphotriesterase-like lactonases (PLL) are the main studied families (Elias and Tawfik, 2012). They share a common catalytic mechanism and their differences in AHL substrate preference mainly lie on how the acyl chain can be accommodated into the catalytic site (Elias and Tawfik, 2012; Bergonzi et al., 2019).

In this study, we investigated the effects of two QQ lactonases with distinct AHL specificities, from PLL and MLL families, on *P. aeruginosa* PA14. We used W263I variant of *Sso*Pox, an engineered variant of a PLL isolated from the thermophilic archaea *Saccharolobus solfataricus* (previously *Sulfolobus solfataricus*) that favors long aliphatic lactones as substrates, such as 3-oxo-C_12_ HSL (Hiblot et al., 2013). Its QQ efficiency for biofilm and virulence factors reduction was previously demonstrated in *P. aeruginosa* with two model strains (PAO1 and PA14) and 51 clinical isolates (Hraiech et al., 2014; Guendouze et al., 2017). This lactonase was also shown to reduce mortality in a rat pneumonia model (Hraiech et al., 2014). The MLL *Gc*L isolated from *Parageobacillus caldoxylosilyticus* which was recently characterized and exhibits broad AHL specificity was chosen as the second lactonase (Bergonzi et al., 2016, 2019; Mahan et al., 2020). *Sso*Pox W263I and *Gc*L, which mainly differ in their ability to hydrolyze C_4_ HSL, were used independently and in combination, to disrupt QS of *P. aeruginosa* PA14 and to investigate the role of lactonase specificity on phenotypes, gene expression and proteome regulation. This work constitutes an extensive molecular and phenotypic comparative study of enzyme-based QQ in *P. aeruginosa* which highlight the importance of lactonase specificity in QQ treatment.

## Results

### Lactonase Specificity on *P. aeruginosa* AHLs

Using an *in vitro* colorimetric assay, the ability of *Sso*Pox W263I and *Gc*L to degrade synthetic *P. aeruginosa* AHLs was first investigated and the kinetic parameters of both enzymes were determined (Table 1). *Gc*L efficiently hydrolyzed both C_4_ and 3-oxo-C_12_ HSL, whereas *Sso*Pox W263I efficiently degraded 3-oxo-C_12_ HSL but exhibited poor activity toward C_4_ HSL as indicated by *k*_cat_/*K*_M_ values. *Sso*Pox W263I is 930 times more specific for 3-oxo-C_12_ than for C_4_ HSL whereas *Gc*L displays catalytic efficiencies (*k*_cat_/*K*_M_) in the same order of magnitude for both substrates. While both enzymes demonstrated similar catalytic efficiencies on 3-oxo-C_12_ HSL, *Sso*Pox W263I showed stronger affinity for this substrate than *Gc*L (lower *K*_M_). Moreover, enzymes strongly diverged regarding their ability to degrade C_4_ HSL by more than two order of magnitude in favor of *Gc*L. At saturating concentration of substrate, *Gc*L and *Sso*Pox showed only a 3-fold difference in specific activities (U.mg^–1^) on 3-oxo-C_12_ HSL, while *Gc*L displayed a 133-fold higher activity on C_4_ HSL as compared to *Sso*Pox W263I in the conditions used for this experiment. Although, it can also degrade C_4_ HSL, *Sso*Pox W263I is mainly active on 3-oxo-C_12_ HSL while *Gc*L can almost equally degrade both substrates. To take into account protein addition in the culture medium, *Sso*Pox variant 5A8, which demonstrated no detectable activity on any AHL (Table 1) was used as a negative control, in the same amounts as active enzymes (Bergonzi et al., 2019).

**TABLE 1:** Kinetic parameters of *Sso*Pox W263I, *Sso*Pox 5A8 and *Gc*L for 3-oxo-C_12_ HSL and C_4_ HSL.

**Enzyme**	***Sso*Pox W263I**		***Gc*L**		***Sso*Pox 5A8**	
**AHL**	**3-oxo-C_12_ HSL**	**C_4_ HSL**	**3-oxo-C_12_ HSL**	**C_4_ HSL**	**3-oxo-C_12_ HSL**	**C_4_ HSL**
*K*_M_ (μM)	40 ± 10	(4.3 ± 0.1) × 10^3^	97 ± 9	(9.8 ± 0.7) × 10^2^	N.D.	N.D.
*k*_cat_ (s^–1^)	2.9 ± 0.1	0.37 ± 0.01	10 ± 1	43 ± 1	N.D.	N.D.
*k*_cat_/*K*_M_ (M^–1^.s^–1^)	(8 ± 2) × 10^4^	86 ± 1	(1.1 ± 0.1) × 10^5^	(4.3 ± 0.3) × 10^4^	N.D.	N.D.
Specificity ratio*	930		2.5		–	
Specific activity (U.mg^–1^)**	4.3 ± 0.5	0.46 ± 0.02	13 ± 2	61 ± 1	N.D.	N.D.
Activity ratio***	–	–	3.0	133	–	–

Values are represented as mean ± SD of *n* = 4 replicates. N.D., not detected. *k_*cat*_/*K*_*M*_ for 3-oxo-C_12_ HSL over *k*_*cat*_/*K*_*M*_ for C_4_ HSL. **Determined at saturating concentration of each AHL. ***Specific activity of *Gc*L over specific activity of *Sso*Pox W263I.

### Both Quorum Quenching Lactonases Similarly Impact AHL Systems but Differently Influence PQS

To decipher the impact of lactonases, mainly differing in their activity toward C_4_ HSL, on the QQ of PA14, *Sso*Pox W263I and *Gc*L were used, alone or in combination, at adjusted concentrations in order to have the same activity regarding the substrate 3-oxo-C_12_ HSL (i.e., equal number of enzymatic Units per volume) in the MOPS minimal medium cultures. PA14 growth in MOPS medium was followed over 26 h. After 20 h, cells in the culture reached stationary phase in all tested conditions (Supplementary Figure 1). Moreover, lactonase activities in the culture medium were not impacted after 20 h, therefore this time point was chosen for further experiments (Supplementary Figure 2).

**FIGURE 1:**
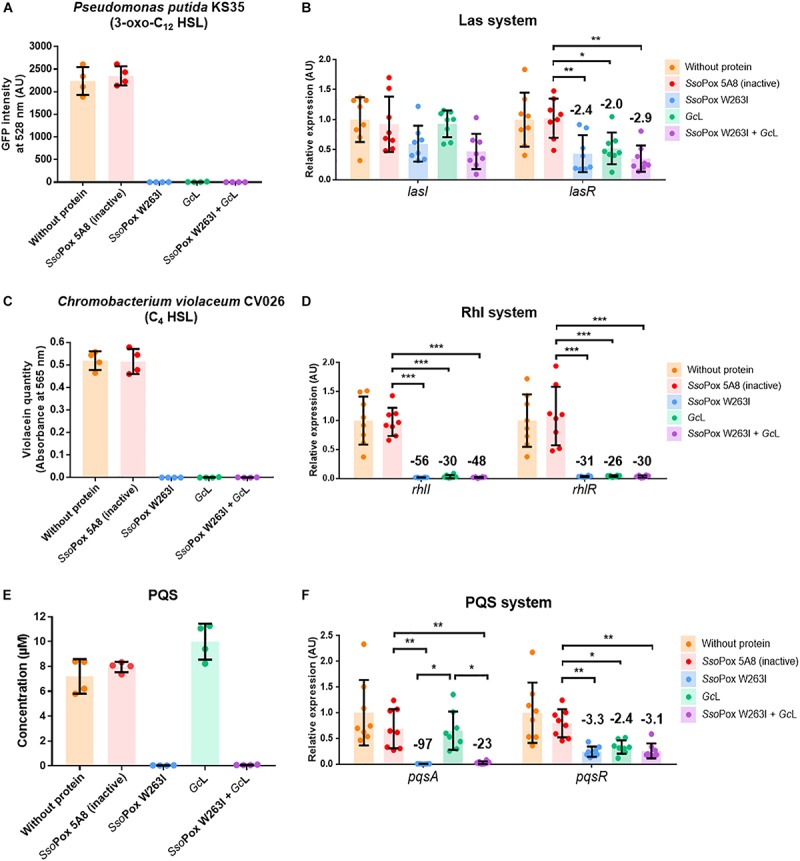
QS molecules and QS gene expression are similarly impacted by the two lactonases except for PQS and its associated synthesis pathway. For each active enzyme or their mixture, 2 U.mL^–1^ activity on 3-oxo-C_12_ HSL was used during culture step. The inactive variant *Sso*Pox 5A8 was used at the same protein quantity as *Sso*Pox W263I. **(A,C,E)** QS molecules measurement after lactonase treatment at 2 U.mL^–1^ on 3-oxo-C_12_ HSL. For each condition, all *n* = 4 independent samples are plotted with mean and standard deviation in colored histogram and black bars. **(B,D,F)** Relative expression of genes involved in Las, Rhl, and PQS systems after lactonase treatment at 2 U.mL^–1^ on 3-oxo-C_12_ HSL. The inactive variant *Sso*Pox 5A8 was added in the same protein quantity as *Sso*Pox W263I. All *n* = 8 independent samples are plotted with mean and standard deviation as colored histograms and black bars. Statistical significance according to Sidak’s multiple comparison test was highlighted by black stars (multiplicity adjusted *p*_value_ < 0.05*, <0.01**, <0.001***). When significantly different from the inactive *Sso*Pox 5A8 control, fold change was added on top of the data.

In order to evaluate the respective impact of both lactonases, alone or in combination, on AHL quantities, the concentration of both C_4_ HSL and 3-oxo-C_12_ HSL in the culture medium was estimated using reporter strains. Surprisingly, although the lactonases were proved to have very distinct specificity toward AHLs, neither C_4_ HSL nor 3-oxo-C_12_ HSL were detectable (Figures 1A,C). To further investigate the potential different role of the lactonases on PA14 regulation, the expression of major QS genes was monitored by RT-qPCR. Consistently with AHL measurements, both enzymes similarly impacted Las and Rhl systems (Figures 1B,D). *lasR* expression was significantly decreased while *lasI* expression was not impacted and the expression of both *rhlI* and *rhlR* was significantly decreased. Nevertheless, strong variations between lactonases were observed regarding PQS system. Although, *pqsR* expression was decreased by both lactonases, a major difference arose for *pqsA*, a gene involved in the synthesis of the PQS autoinducer (Figure 1F). *pqsA* expression was strongly reduced with *Sso*Pox W263I, whereas *Gc*L did not impact *pqsA* transcript levels as compared to the controls. When treated with both enzymes combined, *pqsA* expression followed the same trend as with *Sso*Pox W263I alone with a strong decrease in transcript levels (Figure 1F). To confirm these observations at the molecular level, the amount of 2-heptyl-3,4-dihydroxyquinoline (PQS) was measured in culture supernatants. PQS concentration drastically dropped after treatment with *Sso*Pox W263I alone or combined with *Gc*L as compared to controls and treatment with *Gc*L alone (Figure 1E). Interestingly, the level of 4-hydroxy-2-heptylquinoline (HHQ), a precursor of PQS, was also affected and was mainly detected in culture supernatants of PA14 treated by *Gc*L alone (1.5 μM) as compared to other conditions (<0.15 μM) (Supplementary Figure 3).

Altogether, these results demonstrate that lactonases with distinct AHL specificities similarly impacted the tested AHL-based systems but yielded significant changes in PQS production and regulation. To further probe the impact of these variations on PA14 behavior, phenotypic characterization was performed.

### Both Quorum Quenching Lactonases Inhibit the Production of Virulence Factors *in vitro*

**FIGURE 2:**
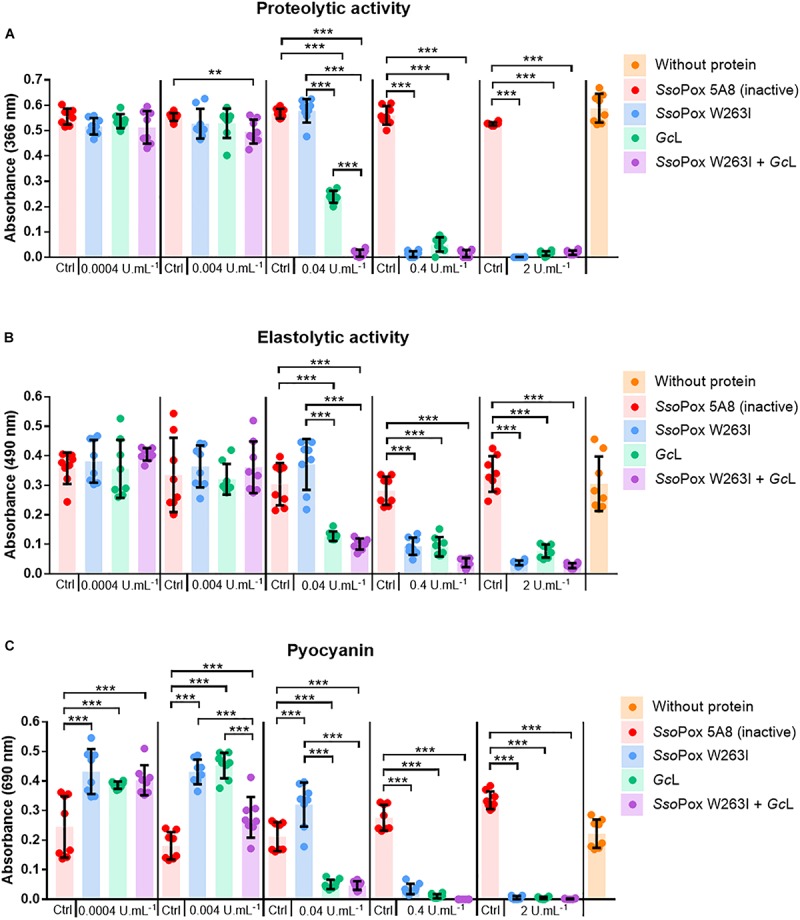
*In vitro* virulence factors are reduced by the two lactonases alone or in combination. **(A–C)** Proteases, elastase B, pyocyanin measurements after lactonase treatment. For each active enzyme or their mixture, an equivalent activity on 3-oxo-C_12_ HSL was used. The inactive variant *Sso*Pox 5A8 was used at the same protein quantity as *Sso*Pox W263I. All *n* = 8 independent samples are plotted with their mean and standard deviation as colored histograms and black bars. Statistical significance according to Holm-Sidak’s multiple comparison test are highlighted by black stars (multiplicity adjusted *p*_value_ < 0.05*, < 0.01**, < 0.001***).

Three representative virulence factors of *P. aeruginosa*, namely pyocyanin, protease and elastase were measured with different enzyme concentrations and combinations. Protease and elastase production decreased with increasing lactonase concentrations, while the addition of inactive variant had no effect (Figures 2A,B). Pyocyanin production slightly increased for the lowest concentrations of enzymes and decreased from 0.04 U.mL^–1^ (Figure 2C). The highest concentration of enzymes reduced the production of elastase by more than 75% and the production of pyocyanin and protease by more than 95% in all conditions. *Gc*L significantly reduced the production of all three virulence factors at lower concentrations than *Sso*Pox W263I (0.04 U.mL^–1^ versus 0.4 U.mL^–1^). Furthermore, the combination of the two lactonases showed combinatory effects and decreased pyocyanin and protease from 0.004 U.mL^–1^ as compared to each enzyme alone (Figures 2A,C).

Reduction of *in vitro* virulence factor production was thus achieved with both enzymes, *Gc*L being efficient at lower concentrations than *Sso*Pox W263I and the combination of both lactonases showed synergistic effect particularly regarding protease inhibition.

### The Two Quorum Quenching Lactonases Have Differential Abilities to Inhibit *in vitro* Biofilm Formation and Biofilm-Associated Tolerance to Antimicrobials

**FIGURE 3:**
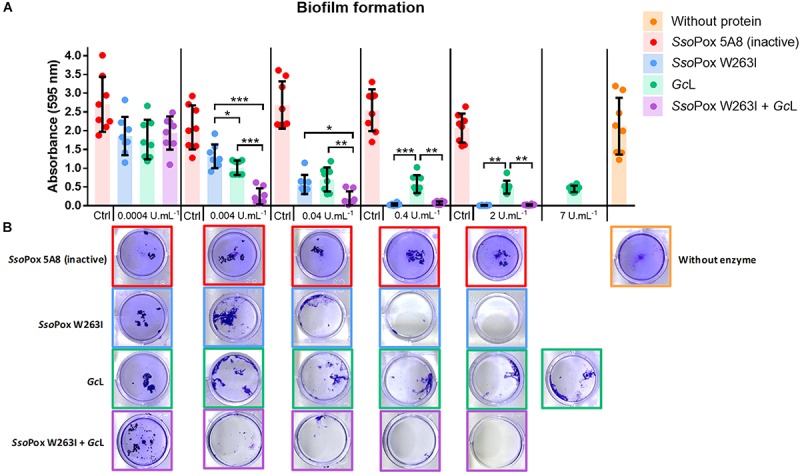
Biofilm formation is reduced by *Gc*L and undetectable with *Sso*Pox W263I. **(A)** Biofilm formation was measured using crystal violet staining. For each active enzyme or their mixture, an equivalent activity on 3-oxo-C_12_ HSL was used. The inactive variant *Sso*Pox 5A8 was used at the same protein quantity as *Sso*Pox W263I. All *n* = 8 independent samples are plotted with their mean and standard deviation as colored histograms and black bars. Statistical significance according to Holm-Sidak’s multiple comparison test are highlighted by black stars (multiplicity adjusted *p*_value_ < 0.05*, < 0.01**, < 0.001***). **(B)** Corresponding pictures of crystal violet stained biofilm after lactonase treatment for each concentration and conditions.

Biofilm formation was reduced in a dose-dependent manner in every condition (Figure 3). However, *Sso*Pox W263I showed a greater anti-biofilm effect than *Gc*L. *Sso*Pox W263I nearly completely inhibited biofilm formation (reduction > 99% for *Sso*Pox W263I and of 76% for *Gc*L at 2 U.mL^–1^) whereas small bacterial aggregates were still observable with *Gc*L even when increasing the concentration up to 7 U.mL^–1^ (Figure 3). The simultaneous utilization of both enzymes showed a combinatory effect at 0.004 U.mL^–1^ as compared to each enzyme alone and inhibited biofilm like *Sso*Pox W263I alone, and unlike *Gc*L, at high concentration.

As biofilm may reduce antimicrobial molecules efficacy, we evaluated the role of QQ to increase bacterial susceptibility to antiseptics and antibiotics. Using the minimal biofilm eradication concentration (MBEC) assay, the sensitivity of PA14 biofilms to antimicrobials following lactonase exposure was determined for tobramycin and gentamicin as well as for H_2_O_2_ (Harrison et al., 2010). In this assay, only adherent cells grown on a peg support are transferred and exposed to antimicrobials. Without antimicrobial application, similar cell quantities were recovered from the peg lid support in every condition (Supplementary Figure 4). However, after pretreatment with *Sso*Pox W263I, PA14 was more sensitive to all three of these antimicrobial agents (Table 2 and Supplementary Figure 4). For PA14 pretreated with *Sso*Pox W263I, the MBEC values were reduced by 10-fold after relatively short exposure periods of 1.5 h to H_2_O_2_ and 3 h for antibiotics. *Gc*L, in contrast, only impacted H_2_O_2_ sensitivity with a 2-fold reduction of the MBEC value (Table 2). The combined pretreatment with both lactonases increased sensitivity for all three antimicrobials with a similar-fold change as *Sso*Pox W263I alone (Table 2 and Supplementary Figure 4). QQ enzymes thus enhanced the efficacy of antimicrobial treatments in PA14. However, *Sso*Pox W263I exhibited much greater ability than *Gc*L (from 5- to 10-fold) to increase antimicrobial sensitivity of *P. aeruginosa* biofilms.

**TABLE 2:** MBEC values for the three antimicrobials tested with lactonases at 2 U.mL**^–^**^1^ on 3-oxo-C_12_ HSL.

**MBEC**	*Sso*Pox 5A8 (inactive)*	***Sso*Pox W263I**	***Gc*L**	***Sso*Pox W263I + *Gc*L**
H_2_O_2_ [mM]	100	10	50	10
Tobramycin [μg.mL^–1^]	10	1	10	1
Gentamicin [μg.mL^–1^]	20	2	20	2

*Equivalent protein quantity as *Sso*Pox W263I condition.

Biofilm formation and associated antimicrobial sensitivity were differently impacted by lactonases. Only *Sso*Pox W263I decreased biofilm formation below detection limit and lowered MBEC values for both antibiotics and H_2_O_2_. Combining both lactonases showed similar results than *Sso*Pox W263I used alone with no synergistic effect observed.

### Both Quorum Quenching Lactonases Reduce Competing Capability of *P. aeruginosa* Toward *Escherichia coli*

**FIGURE 4:**
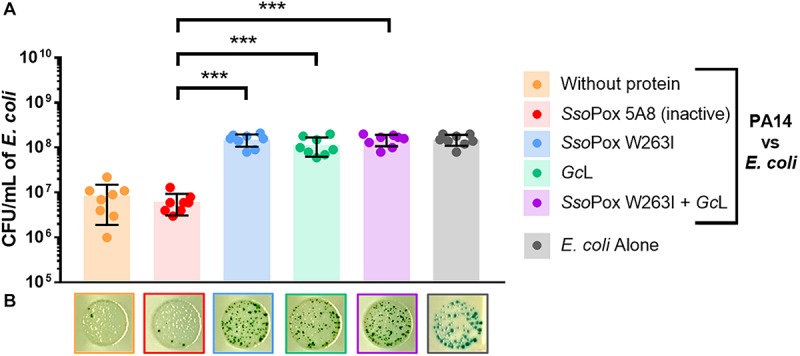
PA14 virulence toward *E. coli* is similarly reduced by both lactonases. **(A)** CFU count of *E. coli* and PA14 recovered after 24 h of incubation. For each active enzyme or their mixture, 2 U.mL^–1^ activity on 3-oxo-C_12_ HSL was used. The inactive variant *Sso*Pox 5A8 was used at the same protein quantity as *Sso*Pox W263I. All *n* = 8 independent samples are plotted with mean and standard deviation as colored histograms and black bars. Statistical significance according to Sidak’s multiple comparison test was highlighted by black stars (multiplicity adjusted *p*_value_ < 0.05*, <0.01**, <0.001***) for *E. coli* count at 24 h. **(B)** Pictures of a representative dilution for each condition where surviving *E. coli* are visible as dark blue colonies.

The capacity of PA14 to compete with other prokaryotes was evaluated using *Escherichia coli* as bacterial competitor over 24 h (Allsopp et al., 2017). In the absence of QQ treatments, PA14 reduced the population of living *E. coli* by more than 10-fold as compared to *E. coli* alone (Figure 4). However, with any QQ treatment at 2 U.mL^–1^, *E. coli* was not killed and a similar number of living cells were recovered as to *E. coli* control (Figure 4). Thus, both QQ lactonases, alone or combined, reduced with similar magnitude the competition of *P. aeruginosa* against another prokaryote, regardless of their substrate specificity.

### Only the Lactonase With Substrate Preference for 3-Oxo-C_12_ HSL Reduces Virulence in an Amoeba Infection Model

**FIGURE 5:**
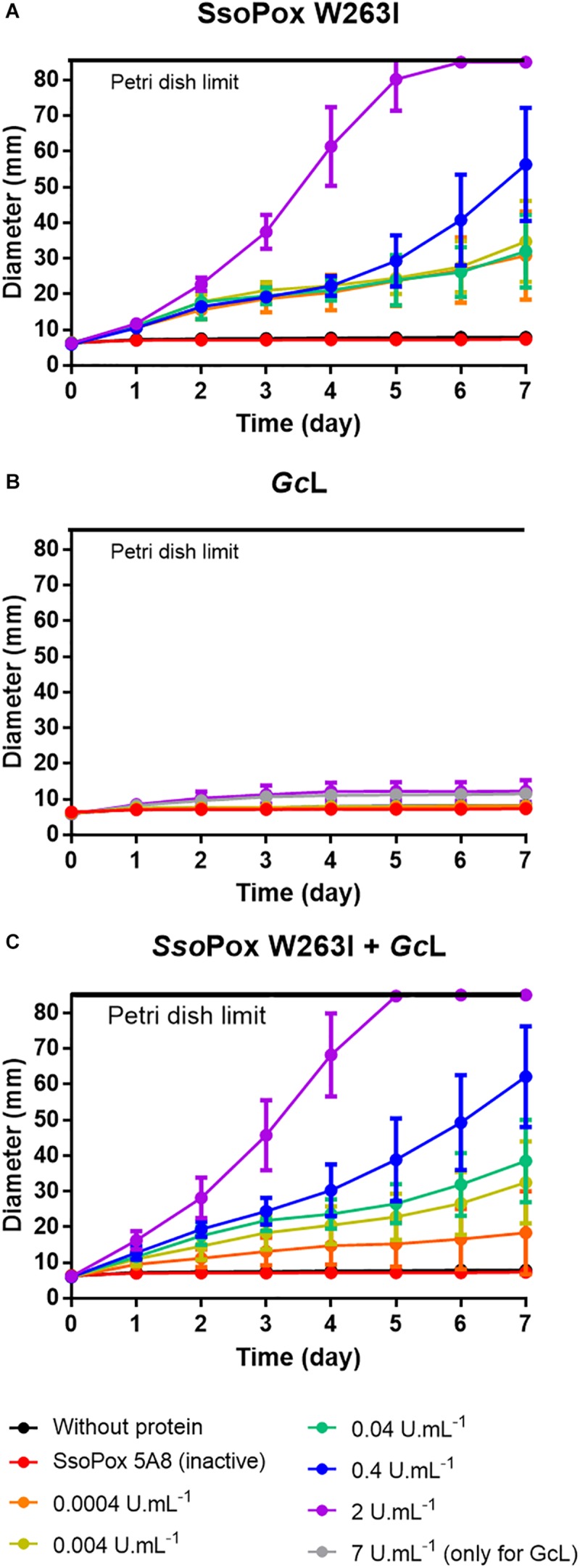
PA14 virulence toward amoeba is only reduced with *Sso*Pox W263I. **(A–C)** Propagation of *A. polyphaga* Linc AP1 in virulence plate assay after treatment of *P. aeruginosa* PA14 by *Sso*Pox W263I, *Gc*L, *Sso*Pox W263I and *Gc*L. All *n* = 8 independent samples are represented by the mean and standard deviation in corresponding colored bars. For each active enzyme or their mixture, an equivalent activity on 3-oxo-C_12_ HSL was used. The inactive variant *Sso*Pox 5A8 was used at the same protein quantity as *Sso*Pox W263I at 2 U.mL^–1^.

To evaluate the QQ impact of both lactonases on PA14 virulence toward a eukaryotic host, an *in vivo* amoeba assay was performed. This assay is based on the ability of *Acanthamoeba polyphaga* Linc AP1 to grow in the presence of quenched or non-quenched bacteria (Fenner et al., 2006; Mion et al., 2019). In both controls, amoebas were not able to grow in presence of PA14 demonstrating its virulence toward *A. polyphaga* (Figure 5). After quenching by *Sso*Pox W263I, virulence toward amoebas was reduced in a dose–response manner and *A. polyphaga* was able to propagate even at the lowest enzyme concentration tested (Figure 5A). Conversely, *Gc*L did not restore the ability of amoebas to grow, even at the highest concentrations (Figure 5B). Finally, when both enzymes were mixed, virulence was reduced to a degree comparable to that of *Sso*Pox W263I alone (Figure 5C). Thus, in this eukaryotic virulence assay, these two lactonases with distinct substrate specificity had different impacts; *Sso*Pox, even when used at 17,500-fold lower concentration, showed greater effects than *Gc*L.

### Treatment of PA14 With Different Lactonases Leads to Distinct Proteomic Profiles

To further investigate the specific effects of signal disruption by the two lactonases and get a broader picture of the differences observed on phenotypes, we conducted proteomic analyses on *P. aeruginosa* PA14 cultures. A total of 515 (8.7%) out of the 5,886 proteins of PA14 were detected and identified (Uniprot database). The relative abundances of 210 proteins were significantly changed (fold change ≥ 2 and *p*_value_ < 0.05) in at least one of the six possible comparisons (Supplementary Dataset S1). This number is in the same range as previous reports investigating QSI effects by proteomic and transcriptomic analyses (Hentzer et al., 2003; Nalca et al., 2006; O’Loughlin et al., 2013; Sethupathy et al., 2016). It provides evidence of the great importance of signal disruption in the regulation of the PA14 proteome. Principal component analysis (PCA) of the 210 proteins resulted in 11 principal components with a cumulative explained variation (*R*^2^X) of 98% and predicted variation (*Q*^2^) of 86% (Supplementary Figure 5). The two first components differentiate three distinct groups accounting for most of the sample variations (cumulated *R*^2^X of 70%) (Supplementary Figures 5A,C). The first principal component highlights the significant difference between the control (*Sso*Pox 5A8) and the three QQ treatments. The second principal component discriminates the treatments using either *Sso*Pox W263I or both enzymes as opposed to *Gc*L alone.

**FIGURE 6:**
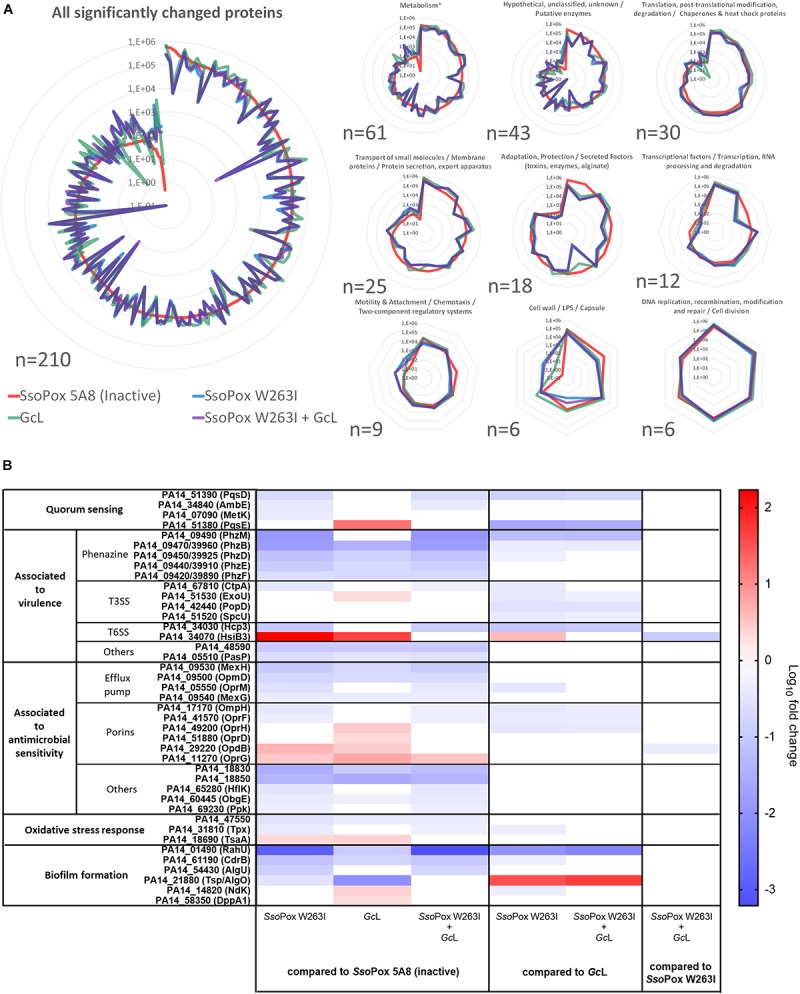
Fluctuations in *P. aeruginosa* PA14 proteomes reflect enzyme-mediated phenotypic changes. For each active enzyme or their mixture, 2 U.mL^–1^ activity on 3-oxo-C_12_ HSL was used. The inactive variant *Sso*Pox 5A8 was used at the same protein quantity as *Sso*Pox W263I. **(A)** Global modifications of PA14 proteome in response to lactonase treatment. The mean of normalized intensities for each condition are plotted. Only the 210 significantly changed proteins were used. The proteins are grouped according to PseudoCAP classification (http://www.pseudomonas.com). ^∗^Metabolism: Amino acid biosynthesis and metabolism/Biosynthesis of cofactors, prosthetic groups and carriers/Carbon compound catabolism/Central intermediary metabolism/Energy metabolism/Fatty acid and phospholipid metabolism/Nucleotide biosynthesis and metabolism. **(B)** Highlighted modifications of PA14 proteome in response to lactonase treatment. Heat map of the log_10_ relative fold change mean for the six comparisons.

The 210 proteins with altered abundance were clustered by biological functions according to PseudoCAP classification (Winsor et al., 2016) (Figure 6A). 29% are associated with metabolic pathways including cofactor, nucleotide or amino acid biosynthesis, as well as the metabolism of energy, carbon compounds, or fatty acids. Proteins related to translation and post-translational modification as well as chaperones and heat shock proteins accounted for 14.3% of altered proteins. Membrane associated proteins represent 11.9% of the impacted proteins and specifically include porins and proteins involved in efflux or protein export. Other affected proteins relate to adaptation, protection and secreted factors (8.6%) and transcriptional regulator or transcription related proteins classes (5.7%). Changes of abundance in proteins involved in motility, attachments and chemotaxis (4.3%), cell wall/LPS/capsule (2.9%) and DNA processing and cell division (2.9%) were also observed. Finally, the remaining impacted proteins (20.5%) were categorized as hypothetical, unclassified, unknown proteins and putative enzymes.

Analyzing fold changes, strong variations were observed between *Gc*L and *Sso*Pox W263I treatments (Figure 6B). The impact of lactonases on QS-involved proteins was first evaluated. Although, LasI/R, RhlI/R, and PqsR proteins were not detected, probably due to concentrations below the detection limit of the method, proteins of the PQS synthesis pathway were detected and their level were strongly altered as a function of the used lactonase. The level of PqsD was only reduced with *Sso*Pox W263I treatment (fold change of −5 and −7 vs. control and *Gc*L respectively) whereas PqsE, an effector protein encoded by the *pqsABCDE* operon but not mandatory for PQS synthesis, was found more abundantly in the proteomic profile of PA14 treated with *Gc*L (fold change of +17 and +44 vs. control and *Sso*Pox W263I) (Drees and Fetzner, 2015; Rampioni et al., 2016).

Several proteins involved in biofilm formation were highlighted by the proteomic analysis. PA14_61190 (PA4624/CdrB), transporter of the adhesin CdrA, was decreased with both lactonases but to a stronger degree with *Sso*Pox W263I as compared to *Gc*L (fold change of −13 and −2 vs. control and *Gc*L) (Borlee et al., 2010; Reichhardt and Wong, 2018). Similarly, the abundance of PA14_01490 (PA0122/RahU), a Rhl activated lipid binding protein, was decreased with both lactonase treatments but to a greater extent with *Sso*Pox W263I (fold change of −959 and −107 vs. control and *Gc*L) (Miklavič et al., 2015). Conversely, *Gc*L induced a stronger decrease of Tsp (or AlgO), a protein part of alginate synthesis regulation, than *Sso*Pox W263I (fold change of −115 and −35 vs. control and *Sso*Pox W263I) (Qiu et al., 2007; Hay et al., 2014; Delgado et al., 2018). However, AlgU, the alginate regulator, and OprF, a pleiotropic porin also involved in biofilm formation, were only significantly reduced with *Sso*Pox W263I (Hay et al., 2014; Chevalier et al., 2017). On the other hand, Ndk, a protein related to alginate synthesis and DppA1, related to aggregation, were both only increased with *Gc*L (Sundin et al., 1996; Kim et al., 1998; Lee et al., 2018).

Several proteins involved in oxidative stress response and antibiotic sensitivity exhibited a modified expression level. The abundance of three proteins (PA14_18690, OpdB, and OprG) were increased and seven others (PA14_47550, PA14_18330/50, ObgE, MexG, MexH, and OmpD) were reduced with both treatments as compared to the control (Aendekerk, 2005; Breidenstein et al., 2008; Dotsch et al., 2009; Balasubramanian et al., 2013; Verstraeten et al., 2015; Sakhtah et al., 2016; Chevalier et al., 2017; Gaviard et al., 2018; Wolloscheck et al., 2018). Several other protein levels showed differences between the two lactonase treatments. The abundance of five proteins (OprF, OprM, HflK, OmpH, and Ppk) were reduced only with *Sso*Pox W263I while two others (OprD and OprH) were increased only with *Gc*L treatment (Fraley et al., 2007; Dotsch et al., 2009; Lister et al., 2009; Hinz et al., 2011; Somprasong et al., 2012; Chevalier et al., 2017; Sanz-García et al., 2019).

Regarding proteins associated with virulence, both lactonase treatments reduced the abundance of PA14_48590 and PA14_05510 (PA0423/PasP in PAO1) (Marquart et al., 2005; Feinbaum et al., 2012). *Sso*Pox W263I and *Gc*L also reduced the levels of phenazine synthesis pathway enzymes PhzB, D, E, F and the known phenazine transporter MexGHI-OpmD as compared to the control (Sakhtah et al., 2016). Interestingly, a stronger reduction was observed with *Sso*Pox W263I than with *Gc*L on PhzB and D, as well as a drastic decrease of PhzM with *Sso*Pox W263I only (fold change of −77 and −14 vs. control and *Gc*L). Changes in protein abundance levels were also observed in type 3 secretion system (T3SS) structural and effector proteins (CtpA, PopD, SpcU, and ExoU), associated with virulence toward amoeba, they were more abundant upon treatment with *Gc*L as compared to *Sso*Pox (Pukatzki et al., 2002; Matz et al., 2008; Seo and Darwin, 2013). Important fluctuations in the abundance of H3 type 6 secretion system (H3-T6SS) proteins, a system which could participate in competition toward eukaryotic and prokaryotic organisms (Lesic et al., 2009; Sana et al., 2013; Jiang et al., 2014), were also detected. The abundance of HsiB3 was higher in lactonase treated samples than in control conditions especially in *Sso*Pox W263I (fold change of +170 and +43 vs. *Sso*Pox W263I and *Gc*L). However, Hcp3 was only reduced with *Sso*Pox W263I treatment (fold change of −10 vs. control).

Altogether these results evidence the large effects of AHL signal disruption using QQ lactonases on the proteome of PA14. Furthermore, these results emphasize that QQ effects between lactonases *Sso*Pox W263I and *Gc*L are distinct while their mixture behaves similarly to *Sso*Pox W263I alone.

## Discussion

In this study, we used two different QQ lactonases, *Sso*Pox W263I that proficiently degrades 3-oxo-C_12_ HSL, but has lower activity toward C_4_ HSL, and *Gc*L, a broad-spectrum lactonase that degrades both lactones with high proficiency. Using these two molecular tools to disrupt the QS circuits of *P. aeruginosa*, we collected phenotypic and molecular evidences that lactonase specificity toward AHLs modulates QQ outputs in *P. aeruginosa.*

First, the effects of both lactonases on PA14 autoinducer concentrations and QS gene expression were investigated. Despite a significant difference in C_4_ HSL hydrolase activity, both enzymes similarly reduced C_4_ HSL and 3-oxo-C_12_ HSL concentrations, consistently the expression levels of *las* and *rhl* genes were comparable with both lactonases. A similar reduction of 3-oxo-C_12_ HSL and Las system is consistent with the fact that enzymes were used at similar activity levels toward this substrate. However, similar reductions of C_4_ HSL and Rhl systems is more surprising as *Sso*Pox W263I is less active than *Gc*L against this AHL. The efficient reduction of C_4_ HSL observed with *Sso*Pox W263I could originate from a slow but sufficient degradation of this AHL throughout the growth of the bacteria leading at the end of the growth to similar results than *Gc*L. Expression of *pqsR* was also reduced with both enzymes suggesting that interfering with AHLs affects other QS systems underlining the interconnection of the three QS systems. We also investigated several pathogenicity-related phenotypes such as pyocyanin, protease, elastase production, biofilm formation and ability to compete. We showed that the lactonase *Gc*L, which is highly proficient against both AHLs, exhibited inhibitory activity at concentrations lower than *Sso*Pox W263I for the production of pyocyanin, protease, and elastase. At concentrations of 0.4 and 2 U.mL^–1^, these three factors were quenched by either enzymes or the combination of both enzymes, evidencing that QQ of these factors is dose-dependent. At low enzyme concentrations, pyocyanin production was increased for all lactonase treatments. This result is consistent with a previous report in which pyocyanin could be induced using a QSI antagonizing RhlR, probably by alleviating RhlR inhibition of PQS system (Welsh et al., 2015). Similarly, low lactonase concentrations could lead to reduced levels of C_4_ HSL that partially alleviate RhlR inhibition on PQS and lead to the induction of pyocyanin production. Pyocyanin was the only tested factor to be induced at low enzyme concentrations. Finally, the competition between PA14 and *E. coli* was also equally affected by any enzymatic treatment with a restoration of *E. coli* survival upon lactonase treatment. These results are supported by the proteomic data which showed that levels of proteins involved in phenazine synthesis and the type VI secretion system were similarly impacted by any enzyme or combination. Thereby, lactonase specificity does not impact QQ of these phenotypes which regulation may mainly rely on AHL based QS systems.

Nevertheless, several major differences arose from the different enzymatic treatments. First *Sso*Pox W263I completely inhibited biofilm formation while cell aggregates were still observable with *Gc*L treatment, including at high enzyme concentrations. Thereby the difference in QQ efficiencies concerning biofilm formation does not solely depend on the AHL hydrolytic activity levels, but lactonase specificity plays an important role for its inhibition. Additionally, *Sso*Pox W263I was more efficient than *Gc*L to increase susceptibility of *P. aeruginosa* PA14 biofilm to H_2_O_2_ and antibiotics. Likewise, in the eukaryotic competition assay, only the treatment with *Sso*Pox W263I decreased PA14 virulence and restored the growth of amoebas. Consistently with phenotypic results, a fraction of the observed variations in PA14 proteomes were largely different as a function of the lactonase substrate specificity. Specifically, distinct regulations occurred in antibiotic resistance, biofilm formation or virulence related proteins echoing alterations observed in phenotypes between lactonase treatments. Regarding QS gene expression levels, a drastic difference arose with the significant and strong reduction of *pqsA* expression in *Sso*Pox W263I treated samples only. This result is consistent with proteomic observations since the levels of PqsD and PqsE, both encoded in the same operon as *pqsA*, were found less abundant in samples treated with *Sso*Pox W263I as compared to *Gc*L. Subsequently, we measured PQS and HHQ concentration levels in culture supernatants and showed that PQS levels were only decreased upon treatment with *Sso*Pox W263I and that its precursor HHQ was increased upon treatment with *Gc*L, suggesting that lactonases differentially impact QS hierarchy. Because PQS is known to contribute to virulence, biofilm and antibiotic susceptibility, variations in PQS concentrations may be involved in the observed differences between lactonase-induced phenotypes (Häussler and Becker, 2008; Lee and Zhang, 2015; Hazan et al., 2016; Maura et al., 2016; Rampioni et al., 2016; Maura and Rahme, 2017). Thereby lactonase specificity differently impacts QS at the molecular level resulting in different phenotypic outputs of QQ.

Unexpectedly, despite exhibiting a narrower substrate specificity spectrum, the lactonase *Sso*Pox W263I inhibited a wider range of virulence-related phenotypes in PA14 than the broad spectrum *Gc*L. In a final set of experiments, combining both enzymes resulted in the same phenotypes, gene expression profiles and proteomes as observed with *Sso*Pox W263I alone. The differences observed in PA14 behaviors following the applied lactonase treatment may be completely explained by AHL substrate preferences, specifically in regards to 3-oxo-C_12_ HSL and C_4_ HSL, of both enzymes. It can also be related to other differences in their catalytic parameters. For example, *Sso*Pox W263I shows a higher affinity for 3-oxo-C_12_ HSL than *Gc*L (i.e., lower *K*_M_ value, meaning that SsoPox W263I is able to reach its maximum rate of reaction at lower concentration of substrate than *Gc*L), and this might modulate the balance between 3-oxo-C_12_ HSL and C_4_ HSL concentrations. It is also possible that some of the observed changes are due to a different, unknown activity of these enzymes since both enzymes are known to be promiscuous. Indeed, both enzymes are known to proficiently hydrolyze various δ- and γ-lactones and to exhibit other low hydrolytic activities against phosphotriesters (both enzymes) and arylesters (*Sso*Pox W263I) (Merone et al., 2005; Hiblot et al., 2013; Bergonzi et al., 2019).

We demonstrated that the specificity of the used QQ lactonase is important to interference strategies: indeed, while both enzymes are capable of decreasing virulence factors and biofilm formation *in vitro*, the magnitude of inhibition and their performances in the tested systems, including *in vivo*, largely vary. Counterintuitively, the lactonase showing the broader activity toward AHLs did not induce the larger QQ impact in PA14. This informs us about the complexity of the interplay between the two AHL QS systems in PA14 and reveals that signal integration by the cell is likely neither linear nor additive. Nevertheless, QQ lactonases constitute promising tools to modulate bacterial communications and associated behaviors including host–bacteria and bacteria–bacteria interactions in complex ecosystems. Lactonases with various specificities should be further assayed on a wider selection of *P. aeruginosa* strains as its QS network is known to vary from one strain to another and to evolve in contact with hosts and other bacteria (Chugani et al., 2012; Feltner et al., 2016; Kostylev et al., 2019; Mahan et al., 2020). Even though QS regulation varies depending on growth conditions and strains, we provide evidence that more than the dose, the choice of enzyme is crucial to maximize QQ strategies.

## Materials and Methods

### Bacterial Strains

For enzyme production, *Escherichia coli* BL21 (DE)_3_-carrying plasmids pGro7/GroEL for chaperones and pET22b with either *Sso*Pox W263I, *Sso*Pox 5A8 (V27G/P67Q/L72C/Y97S/Y99A/T177D/R223L/L226Q/L228M/W263H) or N-terminal strep tagged *Gc*L wild type were used (Bergonzi et al., 2016; Guendouze et al., 2017). *E. coli* E. cloni^®^ 10G transformed with the plasmid pGEM^®^-3Zf(+) (constitutive expression of *lacZ* alpha peptide) was used in the virulence assay. For AHL measurement, the biosensor strains *Pseudomonas putida* KS35 and *Chromobacterium violaceum* CV026 were used (McClean et al., 1997; Steidle et al., 2001). For all the phenotypic and proteomic studies, *Pseudomonas aeruginosa* PA14 (UCBPP-14) was used.

### Enzyme Production and Purification

*Sso*Pox W263I and 5A8 were produced as previously described (Guendouze et al., 2017). Overnight precultures were incubated at 37°C in Luria Bertani (LB) medium (10 g.L^–1^ NaCl, 10 g.L^–1^ tryptone, and 5 g.L^–1^ yeast extract) complemented with chloramphenicol (34 μg.mL^–1^) and ampicillin (100 μg.mL^–1^). Then, cultures in ZYP-5052 medium complemented with the same antibiotics were inoculated and incubated at 37°C until optical density at 600 nm reached 0.8–1 (Studier, 2005). At this state, CoCl_2_ and L-arabinose were added at a final concentration of 0.2 mM and 0.2% (w/v). Cultures were further incubated at 23°C for another 20 h. Afterward, cells were pelleted down by centrifugation (4,400 × *g*, 4°C, 20 min) and resuspended in HEPES lysis buffer (50 mM HEPES, 150 mM NaCl, 0.25 mg.mL^–1^ lysozyme, 0.1 mM phenylmethylsulfonyl fluoride (PMSF), 10 mg.mL^–1^ DNaseI and pH 8.0). The cells were stored overnight at −80°C. The next day, cells were thawed and sonicated three times for 30 s with an amplitude of 45% (QSonica sonicator Q700). Cell debris was pelleted down by centrifugation (12,000 × *g*, 4°C, 30 min) and discarded. The supernatant was heated at 80°C over 30 min to precipitate *E. coli* proteins which were removed afterward by centrifugation (12,000 × *g*, 4°C, 15 min). The remaining proteins were incubated overnight at 4°C in 75% ammonium sulfate in order to precipitate and concentrate *Sso*Pox. After resuspension in HEPES buffer (50 mM HEPES, 150 mM NaCl and pH 8.0), ammonium sulfate was eliminated via desalting (HiPrep 26/10 desalting, GE Healthcare; ÄKTA Avant). The resulting fractions were pooled and concentrated with 10 kDA centricon (Millipore). The proteins were then loaded onto a size exclusion chromatography column (HiLoad 16/600 Superdex^TM^ 75pg, GE Healthcare; ÄKTA Avant) and were eluted in HEPES buffer.

For *Gc*L, cultures were realized in the same conditions, but cells were resuspended in Tris-HCl lysis buffer (50 mM Tris-HCl, 300 mM NaCl, 0.25 mg.mL^–1^ lysozyme, 0.1 mM PMSF and 10 mg.mL^–1^ DNaseI and pH 8.0). After being stored overnight at −80°C, resuspended cells were thawed and sonicated twice for 30 s with an amplitude of 45%. Cell debris was pelleted down by centrifugation (12,000 × *g*, 4°C, 30 min). The crude extract was then loaded onto a Strep-tag column (5 mL StrepTrap HP, GE Healthcare; ÄKTA Avant). The elution was performed in Tris-HCl buffer (50 mM Tris-HCl, 300 mM NaCl and pH 8.0) complemented with 2.5 mM of desthiobiotin (Sigma Aldrich).

After purification on chromatography column, the GcL or SsoPox containing fractions were pooled and concentrated with 30 kDa centricon (Millipore). The purity of each protein was checked by 12.5% SDS-PAGE separation and the concentration was measured using a NanoDrop 2000 spectrophotometer (Thermo Scientific).

### Lactonase Activity Measurement

The activity was measured on (L)-3-oxo-C_12_ and (L)-C_4_ HSL at ambient temperature using a colorimetric pH based assay (Bergonzi et al., 2016). Briefly, lactone ring hydrolysis leads to acidification of the solution which is followed by the absorbance modification of a pH indicator (cresol). For kinetic parameters, the degradation of lactones at different concentrations by each enzyme in a cresol buffered solution (1.25 mM Bicine, 150 mM NaCl, 0.2 mM CoCl_2_, 0.25 mM cresol purple, 3.5% or a minimum of 1.5% DMSO for respectively 3-oxo-C_12_ and C_4_ HSL, and pH 8.3) was followed in 200 μL at 577 nm using a plate reader (SynergyHT, BioTek). 5 μg of *Sso*Pox W263I and 1.6 μg *Gc*L were used on 3-oxo-C_12_ HSL and 50 μg of *Sso*Pox W263I and 1.6 μg of *Gc*L on C_4_ HSL. For specific activity determination, one saturating concentration of AHL was tested (8 mM for C_4_ HSL and 1 mM for 3-oxo-C_12_ HSL). One enzymatic unit (U) corresponds to 1 μmol of substrate hydrolyzed per minute. For *Sso*Pox 5A8, up to 500 μg of protein were used and it proved active toward neither C_4_ nor 3-oxo-C_12_ HSL.

### Culture Media and Conditions

Selected medium and culture conditions were adapted from Welsh and Blackwell (2016). Briefly, *P. aeruginosa* PA14 was precultivated in LB in a 25 cm^2^ culture flask (Corning) and incubated over 5–6 h at 37°C with a 300 rpm agitation (Titramax 3000, Heidolph) to then inoculate cultures at 1/1,000. Cultures were realized in MOPS minimal medium (50 mM MOPS, 4 mM Tricine, 50 mM NaCl, 1 mM K_2_SO_4_, 50 μM MgCl_2_, 10 μM CaCl_2_, 0.3 μM (NH_4_)_6_Mo_7_O_24_, 40 μM H_3_BO_3_, 3 μM Co(OAc)_2_, 1 μM CuSO_4_, 8 μM MnSO_4_, 1 μM ZnSO_4_, pH 7, filter sterilized) complemented with nitrogen (15 mM NH_4_Cl), iron (5 μM Fe_2_SO_4_), phosphate (4 mM K_2_HPO_4_) and carbon (25 mM glutamate) source. Cultures received either *Sso*Pox W263I, *Gc*L or a mixture of both enzymes (50%/50%) to have the same activity (U.mL^–1^) on 3-oxo-C_12_ HSL in all conditions. The inactive variant *Sso*Pox 5A8 was added in a quantity equivalent to *Sso*Pox W263I.

For growth measurement, cells were grown over 26 h at 37°C and 300 rpm agitation. Cell density was estimated by measuring the optical density at 600 nm with a plate reader (SynergyHT, BioTek) and 200 μL of planktonic cells.

### AHL Extraction and Measurement

Acyl-homoserine lactone extraction was performed using a modified method of ethyl acetate liquid-liquid extraction (Elasri et al., 2001). From 1 mL of MOPS bacterial culture, cells were removed by centrifugation (10,000 × *g*, 5 min) and the supernatant was extracted once with ethyl acetate (1:1 v/v). The organic upper phase was evaporated to dryness, and the residues were resuspended into 15 μL of DMSO. As blank, sterile MOPS medium was extracted the same way.

Acyl-homoserine lactones were quantified in the extract using two reporter strains: *P. putida* KS35 and *C. violaceum* CV026. *P. putida* KS35 harbored an integrated transposon carrying a *lasR* gene controlled by a *lac* promoter and a *gfp* gene fused to *las*B promoter which can be activated by LasR binding 3-oxo-C_12_ HSL (Steidle et al., 2001). *C. violaceum* CV026 do not produce AHLs (*cvI* inactivated) but able to detect short-chain AHLs (McClean et al., 1997).

For 3-oxo-C_12_ HSL detection, *P. putida* KS35 was precultivated overnight in LB (supplemented with 50 μg/mL kanamycin) at 30°C and then diluted to 1/10 in 1 mL of fresh LB with 5 μL of extract. After 8 h of culture at 30°C, the fluorescence was measured using a plate reader (SynergyHT, BioTek) with an excitation wavelength of 485 nm and emission detection at 528 nm. For C_4_ HSL, *C. violaceum* CV026 was precultivated overnight in LB at 30°C and then diluted to 1/1,000 in 1 mL of fresh LB with 10 μL of extract. After 24 h of culture at 30°C, the violacein was quantified using a method based on ethyl acetate extraction (Collins et al., 1980). Briefly, 0.5 mL of cell culture was vigorously mixed with ethyl acetate (1:1 v/v) and then 200 μL of the organic upper phase were transferred into a quartz 96 well plate and the OD at 565 nm was measured. Results for each AHL measurement were plotted after background noise removal.

### PQS and HHQ Extraction and Measurement

*Pseudomonas* quinolone signal and HHQ were extracted, as previously described for AHLs, from 1 mL of culture medium with equal volume of ethyl acetate. As blank, sterile MOPS medium was used. After evaporation of the organic phase, the residues were resuspended into 100 μL of HPLC-grade methanol.

For screening and measurement, liquid chromatography coupled to mass spectrometry (LC-MS) was used. Water, methanol and formic acid were ULC-MS grade (Biosolve, Dieuze). Analysis was performed with an Acquity I-Class UPLC chromatography system connected to a Vion IMS Qtof ion mobility-quadrupole-time of flight mass spectrometer (Waters). Samples were maintained at 4°C and randomly injected (5 μL) into a reverse phase column maintained at 35°C (Acquity BEH C18 1.7 μm 2.1 × 50 mm, Waters). Mobile phase flow rate was 0.5 mL.min^–1^ and a composition gradient was set as follows: using water (A) and methanol (B) each containing 0.1% formic acid: 30 to 95% of B (2 min), 95% of B (1 min), initial composition (1 min). Compounds were ionized in the positive mode with a Zspray electrospray ion source: capillary/cone 1.5 kV/20 V, source/desolvation 120/250°C. Ions were monitored using a High Definition MS(E) data independent acquisition method combining a traveling wave ion mobility survey and a tandem MS monitoring (50–1000 m/z, 0.1 s scan time, 6 and 20–30 eV for low and high energy alternate scans, automatic lockmass correction using Leucine Enkephalin at m/z 556.2766). The spectrometer was calibrated beforehand (Major Mix, Waters) to enable Collision Cross Section (CCS) and m/z values measurements. Ion components (retention time, ion mobility drift time and parents/fragments m/z values) were collected from raw data using the UNIFI software (version 1.9.3, Waters). Structures were targeted as follows: 0.2 min retention time window, 2% CCS tolerance (experimental CCS values were 165/167 Å^2^ for HHQ/PQS), 3 ppm m/z tolerance on parent [M + H] + adducts (m/z 244.1696/260.1645 for HHQ/PQS) and 10 mDa m/z tolerance on predicted fragments (including m/z 159.0679/175.06205 for HHQ/PQS). For calibration, HHQ/PQS stock solutions were prepared in methanol from pure standards (>96%; Sigma Aldrich). Culture medium was spiked with both compounds at concentrations ranging from 2 nM to 20 μM for HHQ and from 4 nM to 40 μM for PQS, every 10-fold. The molecules were extracted in the same conditions as previously described and the calibration curve was fitted point to point in order to estimate the concentrations of HHQ/PQS compounds (<15% deviation on controls).

### RNA Extraction and QS Gene Expression Measurement

From 200 μL of culture cells pellet, RNA was extracted and purified with PureLink^TM^ RNA Mini Kit (Invitrogen) and residual DNA were digested with TURBO DNA-*Free*^TM^ kit (Invitrogen). Then cDNA was synthesized using TaqMan^TM^ Reverse Transcription Reagents (Applied Biosystems) and provided random hexamers. Eventually, qPCR was realized using LightCycler^®^ 480 SYBR Green I Master (Roche), and a CFX96 Touch^TM^ Real Time PCR Detection System (BioRad). Primers used are indicated in Supplementary Table S1. The resulting data for each gene were normalized using housekeeping gene *recA* expression and analyzed using the 2^–ΔCt^ method (Schmittgen and Livak, 2008). The results were plotted as relative expression level by dividing each 2^–ΔCt^ values by the mean of the control without added protein.

### Virulence Factors Measurement

Pyocyanin was extracted by mixing 250 μL of chloroform in 500 μL of cell-free supernatant. After centrifugation at 10,000 × *g* for 1 min, 200 μL of the lower chloroform phase were transferred into a quartz 96 well plate. As blank, 200 μL of chloroform were used. The absorbance was measured at 690 nm (Price-Whelan et al., 2007). Results for each condition were plotted after blank absorbance removal.

Protease activity was measured by using azocasein (Sigma Aldrich) degradation assay (Chessa et al., 2000). Briefly, 25 μL of cell-free supernatant were mixed with 675 μL of phosphate saline buffer pH 7.0 and 50 μL of azocasein solution (30 mg.mL^–1^ in water). After 2 h at 37°C with agitation (300 rpm), 125 μL of 20% (w/v) trichloroacetic acid were added. Then, undegraded azocasein was pelleted down by centrifugation (10,000 × *g*, 5 min). Afterward, 200 μL of supernatant were used to measure the optical density at 366 nm. As blank, an equivalent volume of sterile MOPS medium was used. Results for each condition were plotted after blank absorbance removal.

Elastase B activity was measured by using elastin-Congo red conjugate (Sigma Aldrich) degradation assay (Smith et al., 2003). In a 96 well plastic plate (Greiner), 50 μL of cell-free supernatant were mixed with 150 μL of elastin-Congo red solution (5 mg.mL^–1^ in 10 mM Tris-HCl and 1 mM CaCl_2_ buffer at pH 7.2). After 24 h incubation at 37°C with agitation (300 rpm), the plate was left to rest for 10 min at ambient temperature in order to pellet undigested elastin-Congo red. Afterward, 100 μL of the reaction were carefully transferred into an empty well and then absorbance was measured at 490 nm. As blank, an equivalent volume of sterile MOPS medium was used. Results for each condition were plotted after blank absorbance removal.

### Biofilm Formation Measurement

Biofilm was measured using crystal violet (Sigma Aldrich) biomass staining (Hoffman et al., 2005). After culture in 12 well plates (Nunc^TM^, Thermo Scientific), planktonic cells were carefully removed by pipetting. Wells were washed with 3 mL of phosphate buffered saline (PBS) solution (Biomérieux), dried at 37°C and stained with 3 mL of 0.05% (w/v) crystal violet solution. After removing crystal violet, wells were washed with 4 mL of PBS and fixed crystal violet was dissolved with 3 mL of pure ethanol. Using 200 μL, absorbance was measured at 595 nm. As blank, sterile MOPS medium was used in the same culture conditions. Results for each condition were plotted after blank absorbance removal.

### Antimicrobial Sensitivity Assay

The effect of tobramycin, gentamicin and hydrogen peroxide on PA14 was evaluated using the MBEC^TM^ assay (Innovotech) (Harrison et al., 2010). As previously described, PA14 was grown in 180 μL MOPS medium in the 96 well plate of the MBEC^TM^ assay device for 24 h at 37°C under orbital agitation (110 rpm). Afterward, the lid was transferred, first to a 96 well plate containing 190 μL of fresh non-complemented MOPS medium to wash the planktonic cells and then transferred to another plate containing antimicrobial agents. Tobramycin and gentamicin were used at concentrations ranging from 0 to 20 μg.mL^–1^ and H_2_O_2_ from 0 to 500 mM. PA14 was exposed under agitation (110 rpm) for 1.5 h and 3 h to H_2_O_2_ and antibiotics, respectively. The cells were washed in 200 μL of non-complemented MOPS medium and then transferred into 200 μL of recovery LB (LB supplemented with 20.0 g.L^–1^ saponin and 10.0 g.L^–1^ Tween-80). After 1 h, cells were diluted in non-complemented MOPS medium and plated on LB agar. CFU were counted after 3 days growth at 20°C.

### Competition Assay With *E. coli*

Competition between *P. aeruginosa* PA14 and *E. coli* was evaluated using a modified protocol from Allsopp et al. (Allsopp et al., 2017). The *E. coli* strain was cultivated in LB with 100 μg.mL^–1^ of ampicillin in a 25 cm^2^ culture flask and incubated over 5–6 h at 37°C with a 300 rpm agitation to inoculate LB/ampicillin cultures at 1/1,000. After 20 h in 12 well plate, *E. coli* and MOPS cultivated PA14 were harvested from 1.2 mL of culture by centrifugation at 10,000 × *g* for 5 min. The pelleted cells were resuspended into 1.2 mL of PBS and 200 μL were used to evaluate cell density at 600 nm. The remaining 1 mL was centrifuged again, and the cells were resuspended in adequate volume of PBS in order to reach a final OD _600__nm_ of 1 in all samples. *E. coli* was mixed with PA14 or PBS (control) at a volume ratio of 1:5 (corresponding to a cell ratio of approximately 1:10). Then, 20 μL was spotted onto 0.22 μm pore size hydrophilic PDVF membrane (Durapore^®^ GVWP01300, Millipore) resting on a 1.5% agarose plate. After incubation for 24 h at 37°C, cells were recovered by washing the membrane with 200 μL of PBS. Before and after the incubation, cell counts were realized with serial dilutions in PBS plated on LB agar plate complemented with 80 μg.mL^–1^ of 5-bromo-4-chloro-3-indolyl-beta-D-galactopyranoside.

### Virulence Assay Toward Amoeba

The virulence assay using amoeba was adapted from Fenner et al. (2006) and Mion et al. (2019). *Acanthamoeba polyphaga* Linc AP1 was routinely cultivated in peptone yeast extract glucose [20 g.L^–1^ proteose peptone, 2 g.L^–1^ yeast extract, 0.1 M glucose, 4 mM MgSO_4_, 0.53 mM CaCl_2_, 3.4 mM sodium citrate, 50 μM (NH_4_)_2_Fe(SO_4_)_2_, 2.5 mM KH_2_PO_4_, 1.3 mM Na_2_HPO_4_, pH 6.8] medium (Fenner et al., 2006). After 2–3 days of cultivation at 28°C, the cells were pelleted down at 750 g and resuspended in Page’s amoeba saline (PAS) buffer (2 mM NaCl, 16 μM MgSO_4_, 27 μM CaCl_2_, 0.53 mM Na_2_HPO_4_, 1 mM KH_2_PO_4_, pH 6.9). The volume was adjusted to obtain a 10^5^ cells.μL^–1^ final concentration. As for the bacterial growth, after culture in 6 well plates (Nunc^TM^, Thermo Scientific), 3 mL of bacterial culture were pelleted down and resuspended in a minimum of 1 mL of PAS buffer. Depending on their concentration in suspension, the buffer volume was adjusted to have the same concentration of bacteria in each condition. Then a PAS agar plate was flooded with 1 mL of bacterial suspension. After drying at ambient temperature, 5 μL of *A. polyphaga* were spotted at the center and left to dry. Afterward, the plate was incubated at 30°C over 7 days. Each day, amoeba propagation was followed by directly measuring the central spot with a ruler. The results were plotted for each condition from day 0 to 7.

### Protein Extraction

Cells were harvested by centrifugation (10,000 × *g*, 5 min) and washed with 2 mL of PBS and centrifuged again (10,000 × *g*, 5 min). Pellets were resuspended in 100 μL of UTSTS buffer [8 M Urea, 2 M Thiourea, 100 mM NaCl, 25 mM Tris-HCl, pH 8.2 and protease inhibitor (complete, Roche)] and sonicated on ice for 30 s with an amplitude of 15% (Vibra cells) until it became clear. Cell debris was removed by centrifugation (16,000 × *g*, 20 min) and the supernatant was carefully transferred into a dialysis cassette (Slides Alyzer dialysis cassette 2K MWCO, Thermo scientific). The cassette was incubated for 4 h in 2 L with Urea/Ambic buffer (1 M Urea, 50 mM ammonium bicarbonate, pH 7.4) and overnight in 2 L of fresh Urea/Ambic buffer. Protein quantity was estimated with Braford assay (BioRad) and 50 μg of proteins were mixed to Urea/Ambic buffer to a final volume of 50 μL. 1 μL of 0.5 M dithiothreitol in Urea/Ambic buffer was added for the reduction of disulfide bonds and the reaction was conducted over 1 h at 37°C. For alkylation, 2 μL of 0.5 M iodoacetamide in Urea/Ambic buffer were added and let to react over 1 h protected from light. Afterward, pH was checked to be above 7. Protein digestion was performed by adding 2 μL of 1 μg.mL^–1^ trypsin (Agilent) and samples were incubated overnight at 37°C. Digestion efficiency was checked on 10% SDS-PAGE gel. Finally, a detergent removal spin column (Pierce^TM^, Thermo Fisher) and a C18 spin column (Pierce^TM^, Thermo Fisher) were used to clean the samples.

### Label-Free Quantitative Nano-LC-MS/MS Proteomics Analysis

In a first step, protein digests were separated by Ultra Performance liquid chromatography (UPLC) using the NanoAcquity UPLC System (Waters) connected to a Synapt G2Si Q-TOF ion mobility hybrid mass spectrometer (Waters). The chromatographic system was used in 1D configuration with an analytical column (ACQUITY UPLC M-Class Trap Column Reversed-Phase 1.7 μm spherical Hybrid, CSH, 75 μm × 150 mm, Waters) after a trapping column (ACQUITY UPLC M-Class Trap Column Reversed-Phase 5 μm spherical silica, 180 μm × 20 mm, Waters). Eluted peptides were separated using a 100 min gradient (300 nL.min^–1^; 0.5 to 40% acetonitrile–0.1% formic acid). Data-independent MS/MS analysis was performed with the ion mobility feature (HDMSe method). The ion source parameters were: capillary voltage 3 kV, sampling cone voltage 40 V, ion source temperature 90°C, cone gas flow 50 L.h^–1^. Transfer collision low energy was set to 5 V, trap collision low energy was set to 4 V. The high energy ramp was applied from 4 V to 5 V for the trap collision and from 19 V to 45 V for the transfer collision enabling fragmentation of the ions after the ion mobility cell and before the time-of-flight (TOF) MS. On-column sample load was 800 ng (2 μL injected). Each sample was injected in duplicate.

### Proteomic Data Processing and Analysis

The acquired files were imported into Progenesis QI software Version 2.0 (Non-linear Dynamics, Newcastle, United Kingdom) for label-free quantification analysis. The data were aligned automatically and normalized. Processing parameters were 150 counts for the low energy threshold, 30 counts for the elevated energy threshold. The database used to identify peptides contains the protein sequences of *Pseudomonas aeruginosa* PA14 (TrembL, 25/04/2017, 5,886 sequences). Search tolerance parameters were: peptide and fragment tolerance, 15 ppm, FDR < 1%; Minimum Ion matching requirements were three fragments per peptide, seven fragments per protein and two peptides per protein. The enzyme specificity was trypsin allowing 1 missed cleavage, the accepted modifications were carbamidomethyl of cysteine (fixed), oxidation of methionine (variable), carbamyl of lysine and N-terminal (variable), deamidation (variable) of asparagine and glutamine. The protein normalization was performed according to the relative quantitation using non-conflicting peptides. To determine the significance of changes between samples, a significant ANOVA (*p*_value_ < 0.05) and a fold change superior to 2 were used as the thresholds to define differently expressed protein. The mass spectrometry proteomics data have been deposited to the ProteomeXchange Consortium via the PRIDE (Perez-Riverol et al., 2019) partner repository with the dataset identifier PXD017421.

The principal component analysis (PCA) was performed on normalized data using SIMCA 14. The data were Pareto scaled, autofitted for principal components and the Hotelling’s *T*^2^ was used to assess the possible presence of outlier.

For the heat map, the logarithm with base 10 (log_10_) of the fold change was calculated. According to the reference condition, either log_10_ (higher expressed) or – log_10_ (lower expressed) was used in the representation. Non-significant fold changes (*p*_value_ ≥ 0.05 and/or fold change < 2) were considered to have a value equal to 1 and were represented by a zero on the heat map.

### Statistical Analyses

For virulence factors, biofilm, competition assay with *E. coli* and QS gene expression measurement data, statistical analyses were performed using GraphPad Prism 7. The significance level (α), or the probability of committing a type I error, was set at 0.05. For all these data, normality distribution was checked with the D’Agostino and Pearson omnibus normality test.

For virulence factors and biofilm, statistical analyses were performed on raw optical density data (without blank removal). A two-way ANOVA was performed according to enzyme treatment and concentration. Then when ANOVA *p*_value_ was inferior to 0.05, the Holm-Sidak’s multiple comparisons test was used to assess the difference between *Sso*Pox 5A8 (inactive) and *Sso*Pox W263I, *Gc*L or *Sso*Pox W263I + *Gc*L; *Sso*Pox W263I and *Gc*L or *Sso*Pox W263I + *Gc*L; *Gc*L and *Sso*Pox W263I + *Gc*L for each concentration.

For the QS gene expression, statistical analyses were performed on 2^–ΔCt^ (Schmittgen and Livak, 2008). For the competition assay and QS gene expression, a one-way ANOVA was used and if the ANOVA *p*_value_ was inferior to 0.05, the Sidak’s multiple comparison test was used to assess the difference between: without protein and *Sso*Pox 5A8 (inactive); *Sso*Pox 5A8 (inactive) and *Sso*Pox W263I, *Gc*L or *Sso*Pox W263I + *Gc*L; *Sso*Pox W263I and *Gc*L or *Sso*Pox W263I + *Gc*L; *Gc*L and *Sso*Pox W263I + *Gc*L.

## Data Availability Statement

The datasets generated for this study can be found in the ProteomeXchange via the PRIDE database, accession PXD017421.

## Author Contributions

BR, LP, ME, DD, and EC designed the research. BR, LP, NA, and PD performed the research. BR, LP, and DD analyzed the data. DD and EC supervised and coordinated research. BR, LP, ME, DD, and EC wrote the paper.

## Conflict of Interest

ME and EC have a patent WO2014167140 A1 licensed to Gene&GreenTK. LP, DD, BR, ME, and EC report personal fees from Gene&GreenTK during the conduct of the study.

The remaining authors declare that the research was conducted in the absence of any commercial or financial relationships that could be construed as a potential conflict of interest.
