# Supplementary material for: Lactonase Specificity Is Key to Quorum Quenching in Pseudomonas aeruginosa
Source: Front Microbiol. 2020 Apr 24;11:762. doi: 10.3389/fmicb.2020.00762 (PMC7193897; doi:10.3389/fmicb.2020.00762)
Supplement: Supplementary file 2 [file Data_Sheet_2.DOCX]

**Lactonase Specificity is Key to Quorum Quenching in *Pseudomonas aeruginosa***

**Benjamin Rémy^1,2^, Laure Plener^2^, Philippe Decloquement^1^, Nicholas Armstrong^1^, Mikael Elias^3,*^, David Daudé^2,*^, Éric Chabrière^1,*^**

^1^Aix Marseille Univ, IRD, APHM, MEPHI, IHU-Méditerranée Infection, 19-21 Boulevard Jean Moulin, 13005 Marseille, France.

^2^Gene&GreenTK, 19-21 Boulevard Jean Moulin, 13005 Marseille, France.

^3^ University of Minnesota, Department of Biochemistry, Molecular Biology and Biophysics & Biotechnology Institute, St. Paul, MN 55108, USA.

*** Correspondence:**

Prof. Eric Chabrière,

eric.chabriere@univ-amu.fr

Dr. David Daudé,

david.daude@gene-greentk.com

Prof. Mikael Elias,

mhelias@umn.edu

**Supplementary Figure 1.** **Growth curve in log_10_ scale of PA14 in MOPS medium with lactonase treatment.** For each active enzyme or their mixture, 2 U.mL^-1^ activity on 3-oxo-C_12_ HSL was used. The inactive variant *Sso*Pox 5A8 was used at the same protein quantity as *Sso*Pox W263I. All n=4 independent samples are represented by the mean and standard deviation in corresponding colored bars.

**Supplementary Figure 2.** **Lactonase activity on 3-oxo-C_12_ HSL was not impacted during the 20h of culture with PA14.** For each active enzyme or their mixture, 2 U.mL^-1^ activity on 3-oxo-C_12_ HSL was used. The inactive variant *Sso*Pox 5A8 was used at the same protein quantity as *Sso*Pox W263I. For each condition, all n=4 independent samples are plotted with mean and standard deviation in colored histogram and black bars.

**Supplementary Figure 3. HHQ measurement in culture supernatants.** For each active enzyme or their mixture, 2 U.mL^-1^ activity on 3-oxo-C_12_ HSL was used during culture step. The inactive variant *Sso*Pox 5A8 was used at the same protein quantity as *Sso*Pox W263I. For each condition, all n=4 independent samples are plotted with mean and standard deviation in colored histogram and black bars.


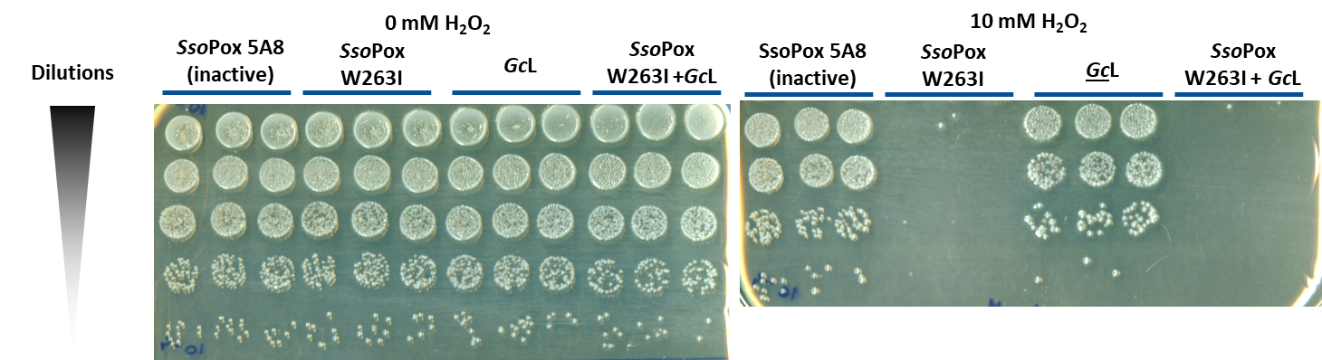


Supplementary Figure 4. Hydrogen peroxide efficiency on PA14 after QQ treatment with each lactonase alone or combined. For each active enzyme or their mixture, 2 U.mL^-1^ activity on 3-oxo-C_12_ HSL was used. The inactive variant *Sso*Pox 5A8 was used at the same protein quantity as *Sso*Pox W263I. Representative results of the CFU counting on agar plate with and without H_2_O_2_ treatment.

Supplementary Figure 5. Principal component analysis (PCA) of each condition according to the 210 changed proteins. (A) Score scatter plot and (B) Loading scatter plot of the two first components. (C) Histogram of the cumulative R2X and Q2 values of the 11 obtained components. The duplicate of the n=4 independent samples of each conditions were used for the PCA analysis.

**Table S1: Primers used in this study.**

| **Primer** | **Sequence 5’-3’** |
| --- | --- |
| lasI_F | AGTGTCATCGACGAGATGGA |
| lasI_R | CTGGAACAGGGTGGTGAAAT |
| lasR_F | TCGGTTATCTGCAACTGCTC |
| lasR_R | GACCCAAATTAACGGCCATA |
| pqsA_F | AACACGCTCGGATTCTGTCG |
| pqsA_R | GGGAATCGAATACAGCCGGT |
| pqsR_F | CAGCGTACTGCTCGACGATT |
| pqsR_R | TTCCGCGTTGTCCTGCTTGA |
| ambB_F | ACGAAGAGCGCCGTTTGCA |
| ambB_R | CCTCGAACAGATGGTGGAGT |
| recA_F | CGCAAGATCACCGGCAATATCA |
| recA_R | GGACCGAGGCGTAGAACTTC |

Dataset S1

Excel table of all detected proteins (Sheet n°1) and the 210 proteins (Sheet n°2) with a fold change ≥ 2 and ANOVA p_value_ < 0.05 for at least one of the six comparisons. For the 210 proteins, the results were color coded for each comparison according to the fold change and p_value_. In some comparisons, PA14_20960 fold change is highlighted in orange and indicated as “infinite” because the protein was undetected in some samples.
